# Association Between Physical Performance and Cognitive Function in Older Adults Across Multiple Studies: A Pooled Analysis Study

**DOI:** 10.1093/geroni/igaa050

**Published:** 2020-10-12

**Authors:** Elizabeth P Handing, Xiaoyan Iris Leng, Stephen B Kritchevsky, Suzanne Craft

**Affiliations:** 1 Department of Internal Medicine, Section on Gerontology and Geriatric Medicine, Wake Forest School of Medicine, Winston-Salem, North Carolina; 2 Department of Biostatistics and Data Science, Wake Forest School of Medicine, Winston-Salem, North Carolina

**Keywords:** Cognition, Diversity, Function/mobility, Measurement

## Abstract

**Background and Objectives:**

While several studies have examined the association between cognitive and physical function, the consistency of these associations across functional contexts is unclear. The consistency of the association between cognitive and physical function performance was examined at baseline across 17 clinical studies with diverse and heterogeneous conditions such as overweight/obese, sedentary, at risk for a mobility disability, osteoarthritis, low vitamin D, or had signs of cognitive impairment.

**Research Design and Methods:**

Data are from 1,388 adults 50 years and older who completed a cognitive and physical function assessment as part of a research study at the Wake Forest Alzheimer’s Disease Research Center or the Wake Forest Older Americans Independence Center. Linear regression models were used to relate cognitive measures (Mini-Mental Status Examination, Montreal Cognitive Assessment, and the Digit Symbol Substitution Task) and physical measures (the Short Physical Performance Battery and hand grip strength) for the whole sample and treat each study as a fixed effect. All models controlled for age, sex, race, and body mass index.

**Results:**

Overall, there was a significant association between higher scores on the Mini-Mental Status Examination (per standard deviation) and better physical function performance (Short Physical Performance Battery score *b* = 0.24, *p* < .001) and its components (gait speed, chair rise, and standing balance; *p*s < .05). Higher scores on the Montreal Cognitive Assessment produced similar results (Short Physical Performance Battery score *b* = 0.31, *p* ≤ .001), and higher scores on the Digit Symbol Substitution Task were also significantly associated with a better Short Physical Performance Battery score (*b* = 0.75, *p* < .001). The relationship between Digit Symbol Substitution Task and physical function performance demonstrated a stronger magnitude of association compared to the Mini-Mental Status Examination or Montreal Cognitive Assessment.

**Discussion and Implications:**

Older adults with heterogeneous health conditions showed a consistent pattern between better cognitive function and better physical function performance with the strongest association among Digit Symbol Substitution Task scores.

**Translational Significance:** This study combining data from 17 diverse clinical studies found a consistent and robust positive relationship between cognitive function and the Short Physical Performance Battery. Specifically, better global cognition (Mini-Mental Status Examination and Montreal Cognitive Assessment) were significantly related to faster walk speed, chair rise, and better balance scores even among older adults who are overweight/obese, sedentary, at risk for a mobility disability, had osteoarthritis, low vitamin D, or had various forms of cognitive impairment. The strongest association was with Digit Symbol Substitution Test scores and chair rise suggesting that psychomotor speed plays an important role in understanding the cognition–physical function relationship.

Numerous studies have examined the relationship between cognitive function and physical function in older adults and describe the relationship as dynamic and bidirectional (1–5). An important missing gap in the current literature is understanding if the relationship differs across populations of older adults with heterogeneous health conditions and if there is a consistent trend across multiple cognitive and physical function measures. The majority of published studies report findings from a single-study population including only one outcome measure of physical functioning. Individual studies lack the ability to make generalization or compare different measures of cognitive and physical function abilities.

For example, gait speed is an inexpensive, simple, reliable, and common measure of three lower extremity physical function among older adults. Slowing in gait speed has been shown to be a valuable signal of potential health concerns including increased risk of disability (6), falls (7), and mortality (8). When paired with memory concerns, it is a strong predictor of cognitive decline (9). However, gait speed is only one aspect of lower extremity function and limited research has examined the association between cognition and other physical function measures.

The Short Physical Performance Battery (SPPB) developed by Guralnik et al. (10) is a widely used, brief clinical test composed of three lower extremity physical function tasks: a 4-m usual gait speed test, a timed chair stand test, and a balance test. The association between cognitive performance and chair stands or balance tests has not been examined as frequently as associations with walking speed, nor has it been investigated in heterogeneous aging populations. Additionally, the relationship between cognitive function and physical performance appears to depend on the assessment measure, and existing literature has shown that the cognitive domain of an executive function is more strongly related to physical function when compared to measures of memory function (11,12).

The purpose of this report is to examine the associations between several cognitive and physical function measures using a pooled analysis approach of 17 clinical studies including older adults with heterogeneous health conditions. Measures of cognition include the Mini-Mental Status Examination (MMSE), Montreal Cognitive Assessment (MoCA), and the Digit Symbol Substitution Test (DSST). Physical function includes the three lower extremity measures collected in the SPPB (4-m walk, chair rise, and balance) as well as hand grip strength. We hypothesize there will be a general trend that better cognitive function will be associated with better physical function across all tasks regardless of health conditions.

## Method

This report uses preexisting data from adults 50 years of age and older from either (a) Wake Forest Alzheimer’s Disease Research Center (WF-ADRC; *n* = 307) or (a) 17 clinical studies conducted through the Wake Forest Claude D. Pepper Older Americans Independence Center (WF-OAIC; *n* = 1115). Each study varies by inclusion criteria and targeted morbidity including those who are overweight/obese, sedentary, at risk for a mobility disability, had osteoarthritis, a diagnosis of acute myeloid leukemia with no cognitive impairment, were listed on a renal transplant list, or had signs of cognitive impairment such as mild cognitive impairment or dementia and were seen at the WF-ADRC. Details of each study can be found in Supplementary Appendix 1. By including studies with diverse levels of physical and cognitive abilities, we are able to examine heterogeneity that typically cannot be explored with detail in a single-site study. The WF-OAIC and WF-ADRC have incorporated the SPPB, MMSE, and MoCA into numerous large clinical studies that allow us to directly compare the measurement across samples of older adults with varying health conditions to understand how broadly the associations between physical and cognitive function may generalize.

### Measures

The current study incorporates previously collected cognitive and physical function data using uniform methodologies that were collected by trained staff across 17 different studies.

#### Self-reported demographics and health status

Participants provided self-reported information about their demographics (age, sex, and medical history). Body mass index (BMI) was measured in these studies and was calculated as kg/m^2^.

#### Physical function

The SPPB (13) consists of three types of physical maneuvers: a timed 4-m usual gait speed test, a timed chair stand test, and a balance test, which are described in detail in the following sections. Each test is scored from 0 to 4, then summed resulting in a total score from 0 to 12. A higher score represents better physical performance.

##### Gait speed

Participants completed a 4-m walk at their usual pace. The location of the walk was on level ground, usually in a corridor, and the distance was specified by tape markings on the ground. Participants completed two walks, and the fastest of the two is used in this analysis.

##### Chair stand

Participants were instructed to fold their arms across their chest and rise five times in a stationary chair without using their arms as quickly as possible. The time to complete the task was recorded in seconds.

##### Balance

The balance test consists of three basic standing foot positions: side-by-side stand, semi-tandem stand, and full-tandem stand (or heel-to-toe). Participants must safely hold the balance position for an allotted amount of time (10 s or less) without assistance. The balance completion is scored from 0 to 4 depending on the completion of holding the standing position.

##### Grip strength

Hand grip strength is a commonly used measure of upper body skeletal muscle function and has been widely used as a general indicator of frailty with predictive validity for both mortality and functional limitation. Hand grip strength was measured in both hands using an adjustable grip strength dynamometer (Jamar Model). Participants performed the test three times with each hand and the maximum overall value was used. Grip strength was available in 13/17 studies.

#### Cognitive function

##### Mini-Mental State Examination

The MMSE (14) is a widely used test of global cognitive function among older adults; it includes tests of orientation, attention, memory, language, and visual–spatial skills. The total possible score is 30 points.

##### Montreal Cognitive Assessment

Some studies in the current project did not administer the MMSE, rather they collected cognitive data using the MoCA (15). It assesses cognitive function in the domains of attention and concentration, executive functions, memory, language, visuospatial skills, conceptual thinking, calculations, and orientation. The total possible score is 30 points.

##### Digit Symbol Substitution Test

The DSST (16) measures psychomotor speed, sustained attention, visual–spatial skills, and set-shifting. The DSST consists of seven rows containing a total of 140 small blank squares, each of which is paired with a randomly assigned number from 1 to 9. Above these rows is a printed key that pairs each number with a different symbol. The participant was asked to fill in the blank spaces with the symbol that is paired to the number as quickly as possible in 120 seconds. The number of correct responses was summed and recorded as the score.

### Design

The current project is a secondary analysis using data from 17 different study populations. Data were collected from participants seen between March 2016 and February 2020 for a baseline visit at the WF-ADRC or who participated in one of 17 different WF-OAIC sponsored clinical studies. All studies were approved by the Institutional Review Board.

### Statistical Analysis

Characteristics of participants for each study were described as mean (standard deviation [SD]) and frequency (percentage) for continuous and categorical variables, respectively. Linear regression models were utilized to examine the relationship between cognitive function (MMSE, MoCA, and DSST) as the predictor variable and physical function (total SPPB score, 4-m walk, chair rise, balance, and grip strength) as the outcome variable. Study was included as a fixed effect and interaction between study and predictor variable was also included to obtain regression coefficients (slope) for each study. A combined model was fitted without the interaction between study and predictor variable to obtain an overall slope. All models were adjusted for age, sex, race, and BMI. To facilitate the comparison across predictors, we standardized different measures of cognitive function to obtain beta coefficients.

Two sets of sensitivity analyses were conducted. First, we restricted the analysis to four studies that had more than one measure of cognitive function (MMSE, MoCA, or DSST scores) to examine the relationship of cognitive function and physical function and compare it with the main analysis; second, we excluded participants from WF-ADRC study diagnosed with mild cognitive impairment or dementia and re-ran the analyses to assess whether this relationship would differ from the main analysis.

## Results

Participant characteristics including age, sex, race, BMI, physical function, and cognitive function scores for participants in each study are described in Table 1. Study participants were predominantly female, white, overweight, and were all ambulatory. Combining all studies, the mean (*SD*) were age = 70 (7) years old, BMI = 31 (5.8), MMSE = 28 (2.3), MoCA = 25 (3.8), and DSST = 54 (15.6). Detailed descriptive statistics for each study are reported in Supplementary Tables 1–3.

**Table 1.** Descriptive Characteristics, Mean (*SD*) or % From 17 Clinical Studies of Older Adults Seen at Wake Forest University

Study Name	*N*	Age	Female %	White %	BMI	SPPB	MMSE	MoCA	DSST
WF-ADRC	289	70.7 (7.9)	69	83	27.7 (5.7)	10.4 (1.8)	27.7 (3.3)	24.0 (4.4)	56.9 (16.0)
APPLE	37	70.4 (3.1)	78	76	35.3 (2.9)	10.9 (1.0)	—	25.3 (2.1)	—
DEMO	116	59.0 (5.4)	100	64	33.5 (3.7)	10.8 (1.0)	29.0 (1.2)	—	—
EVIDNCE	14	74.1 (6.7)	64	36	28.9 (5.3)	8.4 (2.6)	—	22.7 (3.5)	—
HEALTHY	39	69.5 (7.2)	54	97	25.6 (3.9)	11.4 (0.8)	28.6 (1.2)	—	—
IM-FIT	148	70.0 (3.7)	56	86	30.6 (2.3)	10.7 (1.3)	28.3 (1.4)	—	—
INFINITE	195	69.1 (3.6)	72	74	34.6 (3.5)	10.4 (1.5)	28.0 (1.7)	—	—
LEAN	26	70.1 (3.2)	69	100	22.0 (1.7)	11.2 (0.9)	29.2 (1.2)	—	—
MEDFST	138	70.2 (3.7)	73	72	35.4 (3.3)	10.4 (1.5)	—	25.5 (2.7)	56.8 (11.4)
OPTFST	28	70.3 (4.1)	57	61	42.8 (6.0)	10.2 (1.5)	—	24.9 (2.4)	56.9 (13.4)
OPTIMA	87	70.6 (3.6)	46	89	32.7 (5.4)	9.0 (1.0)	28.3 (1.7)	—	—
PA-AML	63	74.0 (7.8)	30	92	28.5 (4.8)	7.6 (3.4)	—	—	36.0 (13.1)
PART	25	65.2 (4.5)	28	64	30.7 (5.4)	9.2 (1.7)	27.1 (2.8)	—	—
PROMO	14	59.0 (5.9)	100	57	31.3 (2.4)	10.1 (1.3)	28.5 (1.4)	—	—
RAINS	70	75.9 (6.0)	63	90	26.9 (4.3)	8.1 (2.1)	27.3 (2.0)	—	—
SILVER	23	68.4 (4.7)	91	61	36.2 (6.2)	11.1 (1.0)	28.1 (1.5)	—	—
SWALLW	76	78.2 (6.8)	51	84	27.7 (4.7)	9.4 (1.8)	26.9 (2.8)	—	51.5 (13.3)

Note: APPLE = Arthritis Pilot for Preserving Muscle While Losing Weight; BMI = Body Mass Index; DEMO = Diet, Exercise, and Metabolism in Older Women; DSST = Digit Symbol Substitution Test; EVIDNCE = Vitamin D Supplementation and Physical Function in Older Adults- Pilot Study; HEALTHY = Database of Determinants of Physical Function in Healthy Older Persons; IM-FIT= Improving Muscle for Functional Independence Trial; INFINITE = Investigating Fitness Interventions in the Elderly; LEAN = Lean Muscle Function; MEDFST = Effect of High Protein Weight Loss on Physical Function for Seniors; MMSE = Mini-Mental Status Examination; MoCA = Montreal Cognitive Assessment; OPTFST = Impact of Weight Loss on Physical Function; OPTIMA = Optimizing Body Composition for Function in Older Adults; PA-ALM = Symptom- Adapted Physical Activity Intervention in Minimizing Physical Function Decline in Older Patients With Acute Myeloid Leukemia Undergoing Chemotherapy; PART = Physical Activity Program for Older Renal Transplant Candidates; PROMO = Dietary Protein & Body Composition in Older Women; RAINS = Reducing Age Related Inflammation with Nutritional Supplementation; SILVER = Use of a Soy-based Meal Replacement Weight Loss Intervention to Impact Ectopic Fat; SD = Standard Deviation; SPPB = Short Physical Performance Battery; SWALLW = CT Imaging of Lingual Muscle Fat Composition in Community-Dwelling Older Adult Aspirators and Non Aspirators. Study descriptions are provided in Supplementary Appendix 1.

**Figure 1.**
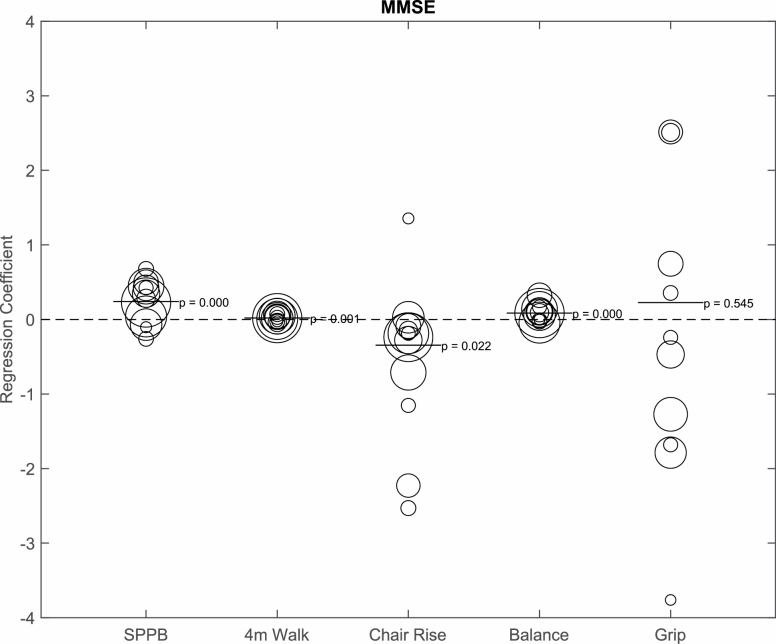
Bubble plot depicting the relationship between Mini-Mental Status Examination (MMSE; per SD) and physical function, adjusted for age, sex, race, and body mass index. The bubble size represents the size of the sample. The bar represents the combined regression coefficient and *p* value. SPPB = Short Physical Performance Battery.

We computed standardized beta coefficients from the linear regression models to examine the association between cognitive function and physical function for each study and for all studies combined. Individual and combined regression coefficients per SD are described in Supplementary Table 4. After adjusting for age, sex, race, and BMI, in the combined analysis for MMSE, a higher score (per SD) on the MMSE was significantly associated with better performance overall on the SPPB (*b* = 0.24, *p* < .001) and specifically with gait speed (*b* = 0.02, *p* < .001), chair rise (*b* = −0.34, *p* = .008), and balance (*b* = 0.09, *p* < .001; Figure 1). MMSE was not significantly related to grip strength (*b* = 0.23, *p* = .54). The study with a strong association between MMSE and physical performance was the Reducing Age-related Inflammation with Nutritional Supplementation (RAINS) study. Results are SPPB (*b* = 0.50, *p* = .01), gait speed (*b* = 0.03, *p* = .24), chair rise (*b* = −2.23, *p* < .001), balance (*b* = 0.32, *p* < .001), and grip strength (*b* = 2.51, *p* = .01).

**Figure 2.**
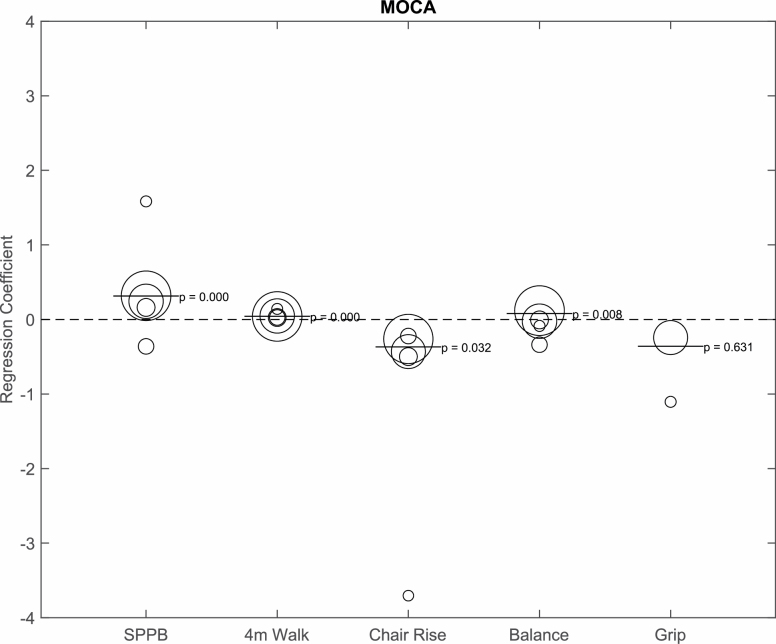
Bubble plot depicting the relationship between Montreal Cognitive Assessment (MoCA; per SD) and physical function, adjusted for age, sex, race, and body mass index. The bubble size represents the size of the sample. The bar represents the combined regression coefficient and *p* value. SPPB = Short Physical Performance Battery.

The MoCA was administered in five studies (Figure 2). Fully adjusted combined analyses showed a significant positive relationship between MoCA (per SD) and physical function measures: overall SPPB score (*b* = 0.31, *p* < .001), gait speed (*b* = 0.04, *p* < .001), chair rise (*b* = −0.37, *p* = .032), and balance (*b* = 0.08, *p* = .008). Grip strength and MoCA were not significantly related (*b* = −0.36, *p* = .63). Individually, the EVIDNCE study (a pilot study of vitamin D supplementation and physical function in older adults) depicted a surprisingly strong association between MoCA and chair rise (*b* = −3.70, *p* ≤ .001) and grip strength (*b* = −1.10, *p* = .57), although grip strength was not statistically significant.

**Figure 3.**
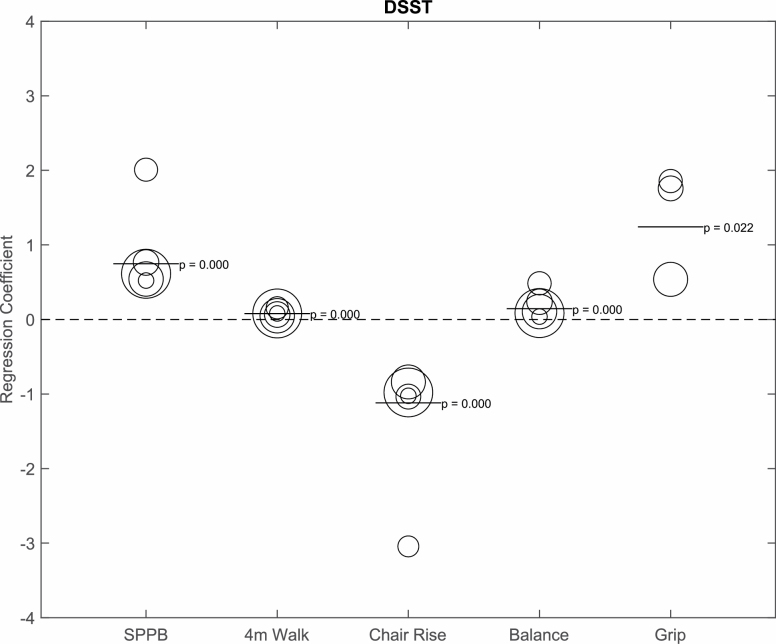
Bubble plot depicting the relationship between Digit Symbol Substitution Test (DSST; per SD) and physical function, adjusted for age, sex, race, and body mass index. The bubble size represents the size of the sample. The bar represents the combined regression coefficient and *p* value. SPPB = Short Physical Performance Battery.

Lastly, in combined analyses with full adjustments, higher scores on the DSST (per SD) were significantly associated with better performance in overall SPPB score (*b* = 0.75, *p* < .001), gait speed (*b* = 0.08, *p* < .001), chair rise (*b* = −1.12, *p* < .001), balance (*b* = 0.14, *p* < .001), and grip strength (*b* = 1.24, *p* = .022) as shown in Figure 3. The study depicted as the strongest association with DSST and physical performance was the PA-AML study (older patients with acute myeloid leukemia). Results are SPPB (*b* = 2.01, *p* < .001), gait speed (*b* = 0.16, *p* < .001), chair rise (*b* = −3.04, *p* < .001), balance (*b* = 0.48, *p* < .001), and grip strength (*b* = 1.86, *p* = .72).

In a sensitivity analysis of four studies that included more than one cognitive test, the DSST–SPPB relationship (*b* = 0.57, *p* < .001) was nearly twice as strong as the MMSE–SPPB (*b* = 0.25, *p* < .001) or MoCA–SPPB relationship (*b* = 0.29, *p* < .001). Results are given in Supplementary Table 5. A second sensitivity analysis was conducted on only cognitively normal participants, excluding participants from the WF-ADRC with mild cognitive impairment or dementia. Results did not significantly differ from the main analysis although the DSST–SPPB relationship was strengthened. Results are MMSE–SPPB (*b* = 0.16, *p* < .001), MoCA-SPPB (*b* = 0.16, *p* = .046), and DSST–SPPB (*b* = 0.75, *p* < .001).

## Discussion

In this study, we examined associations between cognitive function and physical function from 17 different clinical studies of older adults with various health conditions/diseases. We were able to decompose the SPPB into its subtests, which provides more details about physical function performance beyond traditional measures of gait speed or a composite score. Our study demonstrated that in combined analyses, better cognitive function as measured by the MMSE, MoCA, and DSST scores was significantly associated with better physical performance in gait speed, chair rise, and balance independent of age, gender, race, BMI, and comorbidities. Our results add to the increasing evidence that physical function and cognitive function are significantly related (1–5) and add new insights that this relationship remains robust even among older adults who are overweight/obese, sedentary, at risk for a mobility disability, have osteoarthritis, low vitamin D, or have various forms of cognitive impairment.

In specific studies, such as the RAINS study, which had an average age of 76, average BMI = 26, and had lower physical function scores (means: SPPB = 8, gait speed = 0.9 m/s, MMSE = 27, and DSST = 57), there was a significant relationship between overall SPPB (*b* = −2.23) and grip strength (*b* = 2.51). In comparison, in the DEMO study (average age = 59, 100% female, average BMI = 34), this study had higher physical function scores (means: SPPB = 11, gait speed = 1.2 m/s, MMSE = 29); however, there was not a significant cognitive–physical function relationship. This highlights the heterogeneity across studies and how the cognitive–physical function relationship differs among subgroups of people. Other comparisons can be made across studies that varied by health conditions (i.e., HEALTHY vs PA-AML), sex (female-only studies: DEMO and PROMO), race, or cognitive status (WF-ADRC). Longitudinal data are needed to examine these effects in more detail in order to explain possible mechanisms related to the cognition–physical function relationship.

Many of the studies included in our analysis involved older adults who were overweight or obese. Research from combined data of 13 clinical studies found that higher BMI is related to lower SPPB scores and gait speed (17). Findings are mixed regarding the role of BMI and cognition, but our study shows that independent of age, sex, race, and BMI, our results found a significant cognitive–physical function relationship across various tasks.

Consistent with other research, DSST has been shown to be more strongly related to gait and mobility when compared to domains of memory or global cognition (1,18,19). The DSST is a complex cognitive task that involves cognitive abilities such as psychomotor speed, working memory, attention, set-shifting and recruits brain resources in the frontal lobes (20,21). The frontal and prefrontal cortex have also been posited to be recruited when completing a physical function task such as walking or standing from a chair, suggesting there is a shared mechanism between cognitive and physical function (22,23). Our results including adults with comorbidities showed a significant relationship between DSST and physical function performance (gait speed, chair raise, balance, and grip strength). The study with the strongest DSST–physical function association was seen in the PA-AML study (older adults with acute myeloid leukemia). This could be clinically relevant and important for patients with acute myeloid leukemia, highlighting the role of psychomotor speed and better physical function.

Of note, results on the MMSE and MoCA were comparable and did not significantly differ in the association with physical function performance. In the sensitivity analysis of four studies which included two or more cognitive measures, the beta coefficients were very similar to the main analysis showing that the DSST represented the strongest association among cognitive tasks. More research is needed to understand the compatibility between these three measures because there are advantages and disadvantages to each measure.

An important strength and novelty of this report is the ability to compare diverse study populations including a broad range of older adults who are well-functioning or impaired in the domains of cognitive and physical function. Pooling data together from 17 clinical studies we were able to compare the same uniform measurements across studies, which provides results that are more generalizable and is otherwise not possible in a small individual study. To the best of our knowledge, this is the first study to compare the association between cognitive and physical function across different domains of function and by different assessment types among older adults with varying health conditions.

The limitations of our data are that the associations are cross-sectional, therefore we cannot determine the mechanisms or evaluate if abilities declined or changed over time. Some of our measures, such as MoCA and grip strength, were collected in fewer of the studies (5/17 and 11/17 studies). Other health-related data such as socioeconomic status, physical activity, education level, diet, or sleep quality were not uniformly collected across studies, therefore we were not able to evaluate their contribution. Future research should evaluate the role of lifestyle habits and its association with cognitive and physical function performance, as these factors may provide additional insights into the cognitive–physical function relationship. Additionally, future longitudinal research should explore the temporal association between different cognitive function and physical function tasks so as to provide more information on the sequence and change in these abilities among older adults with heterogeneous conditions.

## Conclusions

Understanding the relationship between cognition and mobility may be beneficial for understanding a person’s ability to remain independent. Higher MMSE and DSST scores were significantly associated with higher SPPB scores in combined analyses of 17 clinical studies. Our results provide evidence that the cognition–physical function association remains robust even in a sample of heterogeneous older adults and varying levels of function.

## Supplementary Material

igaa050_suppl_Supplementary-Material
