# Supplementary material for: Association Between Physical Performance and Cognitive Function in Older Adults Across Multiple Studies: A Pooled Analysis Study
Source: Innov Aging. 2020 Oct 12;4(6):igaa050. doi: 10.1093/geroni/igaa050 (PMC7679973; doi:10.1093/geroni/igaa050)
Supplement: igaa050_suppl_Supplementary-Material [file igaa050_suppl_supplementary-material.docx]

**Online Supplementary Material for Accepted Publication in *Innovation in Aging*:**

Association between physical performance and cognitive function in older adults across multiple studies: A pooled analysis study

Elizabeth P. Handing, PhD^1,*^, Xiaoyan Iris Leng, MD, PhD^2^, Stephen B. Kritchevsky, PhD^1^, Suzanne Craft, PhD^1^

1. Department of Internal Medicine, Section on Gerontology and Geriatric Medicine, University of Wake Forest, School of Medicine, Winston-Salm, North Carolina
2. School of Medicine, Department of Biostatistics and Data Science, University of Wake Forest, Winston-Salm, North Carolina

*Address correspondence to: Elizabeth P. Handing, PhD, 1 Medical Center Blvd, Winston-Salem, NC 27157. E-mail: ehanding@wakehealth.edu

Supplementary Table 1. Mean, Standard Deviation (SD), Median, [Minimum, Maximum] for Age and Body Mass Index (BMI) from 17 clinical studies at Wake Forest University

|  | **Age** | | | **BMI** | | |
| --- | --- | --- | --- | --- | --- | --- |
| Study Name | Mean | SD | Median | Mean | SD | Median |
| WF-ADRC | 70.7 | 7.9 | 71.0 [55,90] | 27.7 | 5.7 | 26.7 [16,52] |
| APPLE | 70.4 | 3.1 | 69.9 [66,80] | 35.3 | 2.9 | 35.1 [30,41] |
| DEMO | 59.0 | 5.4 | 58.4 [50,71] | 33.5 | 3.7 | 33.0 [24,41] |
| EVIDNCE | 74.1 | 6.7 | 74.6 [65,87] | 28.9 | 5.3 | 29.2 [22,40] |
| HEALTHY | 69.5 | 7.2 | 68.4 [60,84] | 25.6 | 3.9 | 26.0 [19,37] |
| IM-FIT | 70.0 | 3.7 | 69.4 [65,80] | 30.6 | 2.3 | 30.3 [27,35] |
| INFINITE | 69.1 | 3.6 | 68.0 [65,80] | 34.6 | 3.5 | 33.8 [30,45] |
| LEAN | 70.1 | 3.2 | 69.6 [65,76] | 22.0 | 1.7 | 22.6 [18,24] |
| MEDFST | 70.2 | 3.7 | 69.4 [65,79] | 35.4 | 3.3 | 35.1 [30,42] |
| OPTFST | 70.3 | 4.1 | 68.8 [65,80] | 42.8 | 6.0 | 41.4 [35,64] |
| OPTIMA | 70.6 | 3.6 | 70.2 [65,80] | 32.7 | 5.4 | 31.7 [24,49] |
| PA-AML | 74.0 | 7.8 | 72.9 [60,96] | 28.5 | 4.8 | 28.5 [19,41] |
| PART | 65.2 | 4.5 | 64.5 [60,74] | 30.7 | 5.4 | 29.2 [23,45] |
| PROMO | 59.0 | 5.9 | 58.4 [51,71] | 31.3 | 2.4 | 31.0 [27,35] |
| RAIN | 75.9 | 6.0 | 75.9 [65,86] | 26.9 | 4.3 | 26.5 [20,48] |
| SILVER | 68.5 | 4.7 | 68.0 [61,78] | 36.2 | 6.2 | 35.6 [28,53] |
| SWALLW | 78.2 | 6.8 | 80.0 [64,90] | 27.6 | 4.7 | 26.9 [18,43] |

Note. Study name abbreviations are found in Appendix 1, SD= standard deviation

Supplementary Table 2. Mean, Standard Deviation (SD), Median, [Minimum, Maximum] for Physical Function Tests from 17 clinical studies at Wake Forest University

|  | **SPPB** | | | **4m walk, (m/s)** | | | **Chair rise, (s)** | | | **Balance score** | | | **Grip strength (kg)** | | |
| --- | --- | --- | --- | --- | --- | --- | --- | --- | --- | --- | --- | --- | --- | --- | --- |
| Study Name | Mean | SD | Median | Mean | SD | Median | Mean | SD | Median | Mean | SD | Median | Mean | SD | Median |
| WF-ADRC | 10.4 | 1.8 | 11.0 [2,12] | 1.0 | 0.2 | 1.0 [0.3,2.1] | 12.4 | 3.6 | 12.0 [5.7,36.4] | 3.8 | 0.7 | 4.0 [0,4] | - | - | - |
| APPLE | 10.9 | 1.0 | 11.0 [8,12] | 1.0 | 0.1 | 1.0 [0.7,1.2] | 12.1 | 2.5 | 11.5 [6.5,17.0] | 3.9 | 0.3 | 4.0 [3,4] | - | - | - |
| DEMO | 10.8 | 1.0 | 11.0 [8,12] | 1.2 | 0.2 | 1.2 [0.8,1.8] | 13.6 | 5.7 | 12.4 [8.3,66.1] | 4.0 | 0.2 | 4.0 [2,4] | 31.3 | 6.5 | 31.0 [13.0,49.5] |
| EVIDNCE | 8.4 | 2.6 | 9.0 [4,12] | 0.8 | 0.2 | 0.8 [0.5,1.2] | 18.2 | 5.6 | 17.5 [8.5,27.9] | 3.3 | 1.1 | 4.0 [1,4] | 27.1 | 7.8 | 26.0 [17.0,41] |
| HEALTHY | 11.4 | 0.8 | 12.0 [9,12] | 1.2 | 0.2 | 1.3 [0.9,1.5] | 10.7 | 2.8 | 10.9 [4.0,19.9] | 4.0 | 0.2 | 4.0 [3,4] | 36.6 | 11.8 | 34.5 [18.5,62.5] |
| IM-FIT | 10.7 | 1.3 | 11.0 [7,12] | 1.1 | 0.2 | 1.1 [0.7,1.7] | 12.4 | 3.4 | 12.3 [5.5,23.5] | 3.8 | 0.5 | 4.0 [1,4] | 31.4 | 11.5 | 28.5 [10.0,69.0] |
| INFINITE | 10.4 | 1.5 | 11.0 [3,12] | 1.0 | 0.2 | 1.0 [0.5,1.4] | 13.3 | 4.1 | 12.6 [5.4,31.4] | 3.8 | 0.5 | 4.0 [0,4] | - | - | - |
| LEAN | 11.2 | 0.9 | 11.0 [9,12] | 1.2 | 0.2 | 1.3 [0.8,1.8] | 11.6 | 2.9 | 11.2 [7.2,20.6] | 4.0 | 0.0 | 4.0 [4,4] | 30.4 | 8.2 | 28.5 [20.0,52.5] |
| MEDFST | 10.4 | 1.5 | 11.0 [5,12] | 1.0 | 0.2 | 1.0 [0.5,1.6] | 12.7 | 3.2 | 12.3 [7.2,29.7] | 3.9 | 0.5 | 4.0 [1,4] | 26.5 | 9.1 | 25.0 [15.0,57.0] |
| OPTFST | 10.2 | 1.5 | 11.0 [6,12] | 1.0 | 0.2 | 1.0 [0.6,1.3] | 13.4 | 3.2 | 12.8 [7.4,20.2] | 3.8 | 0.6 | 4.0 [2,4] | - | - | - |
| OPTIMA | 9.0 | 1.0 | 9.0 [6,10] | 1.0 | 0.2 | 1.0 [0.5,1.4] | 17.7 | 5.3 | 16.5 [11.3,53.9] | 3.6 | 0.7 | 4.0 [1,4] | 33.2 | 10.1 | 31.0 [13.5,53.0] |
| PA-AML | 7.6 | 3.4 | 9.0 [0,12] | 0.8 | 0.3 | 0.8 [0.3,1.4] | 15.0 | 4.7 | 14.6 [12.0,26.5] | 3.0 | 1.4 | 4.0 [0,4] | 31.0 | 10.6 | 31.0 [12.0,62.0] |
| PART | 9.2 | 1.7 | 10.0 [5,12] | 0.9 | 0.2 | 0.9 [0.6,1.3] | 14.7 | 2.7 | 14.6 [9.3,22.0] | 3.6 | 0.7 | 4.0 [2,4] | 34.3 | 10.6 | 35.0 [15.0,50.0] |
| PROMO | 10.1 | 1.3 | 10.0 [8,12] | 1.0 | 0.2 | 1.0 [0.8,1.3] | 14.9 | 5.1 | 14.4 [3.5,23.5] | 4.0 | 0.0 | 4.0 [4,4] | 27.4 | 5.5 | 27.5 [18.0,36.5] |
| RAINS | 8.1 | 2.1 | 9.0 [1,12] | 0.9 | 0.2 | 0.9 [0.3,1.4] | 18.8 | 9.2 | 16.7 [9.0,73.9] | 3.1 | 1.2 | 4.0 [0,4] | 31.3 | 12.4 | 28.0 [12.0,65.0] |
| SILVER | 11.1 | 1.0 | 11.0 [8,12] | 1.0 | 0.2 | 1.0 [0.7,1.4] | 11.5 | 2.6 | 11.5 [7.8,19.4] | 3.9 | 0.3 | 4.0 [3,4] | 28.8 | 8.5 | 27.0 [15.0,53.0] |
| SWALLW | 9.4 | 1.8 | 9.5 [4,12] | 1.0 | 0.2 | 1.0 [0.6,1.5] | 14.5 | 3.2 | 14.5 [5.5,21.4] | 3.4 | 0.9 | 4.0 [1,4] | 30.9 | 11.6 | 30.0 [12.0,60.0] |

Note. Study name abbreviations are found in Appendix 1, SD= standard deviation, SPPB= Short Physical Performance Battery scored 0-12, m/s= meters per second, s= seconds, a balance score of 0 is a valid number, not a missing value. All balance score are from 0-4.

Supplementary Table 3. Mean, Standard Deviation (SD), Median, [Minimum, Maximum] for Cognitive Function Tests from 17 clinical studies at Wake Forest University

|  | **MMSE** | | | **MoCA** | | | **DSST** | | |
| --- | --- | --- | --- | --- | --- | --- | --- | --- | --- |
| Study Name | Mean | SD | Median | Mean | SD | Median | Mean | SD | Median |
| WF-ADRC | 27.7 | 3.3 | 29.0 [1,30] | 24.0 | 4.4 | 25.0 [6,30] | 56.9 | 16.0 | 56.5 [1,107] |
| APPLE | - | - | - | 25.3 | 2.1 | 25.0 [22,29] | - | - | - |
| DEMO | 29.0 | 1.2 | 29.0 [25,30] | - | - | - | - | - | - |
| EVIDNCE | - | - | - | 22.7 | 3.5 | 22.0 [19,30] | - | - | - |
| HEALTHY | 28.6 | 1.2 | 29.0 [24,30] | - | - | - | - | - | - |
| IM-FIT | 28.3 | 1.4 | 29.0 [22,30] | - | - | - | - |  | - |
| INFINITE | 28.0 | 1.7 | 28.0 [23,30] | - | - | - | - | - | - |
| LEAN | 29.2 | 1.2 | 30.0 [26,30] | - | - | - | - | - | - |
| MEDFST | - | - | - | 25.5 | 2.7 | 26.0 [18,30] | 56.8 | 11.4 | 56.0 [33,87] |
| OPTFST | - | - | - | 24.9 | 2.4 | 25.0 [20,30] | 56.9 | 13.4 | 57.0 [30,84] |
| OPTIMA | 28.3 | 1.7 | 29.0 [22,30] | - | - | - | - | - | - |
| PA-AML | - | - |  | - | - | - | 36.0 | 13.1 | 36.0 [7,65] |
| PART | 27.1 | 2.8 | 28.0 [18,30] | - | - | - | - | - | - |
| PROMO | 28.5 | 1.4 | 29.0 [26,30] | - | - | - | - | - | - |
| RAINS | 27.3 | 2.0 | 28.0 [20,30] | - | - | - | - | - | - |
| SILVER | 28.1 | 1.5 | 29.0 [25,30] | - | - | - | - | - | - |
| SWALLW | 26.9 | 2.8 | 28.0 [15,30] | - | - | - | 51.4 | 13.3 | 53.0 [15,83] |

Note. Study name abbreviations are found in Appendix 1, SD= standard deviation, MMSE= Mini Mental Status Exam, DSST= Digit Symbol Substitution Test, MoCA= Montreal Cognitive Assessment

Supplementary Table 4. Regression Coefficient (SD unit) Adjusted for Age, Sex, Race, and Body Mass Index, Combined Total and By Each Study

|  |  | **SPPB** |  | **4m walk (m/s)** |  | **Chair rise (s)** |  | **Balance score** |  | **Grip strength (kg)** |  |
| --- | --- | --- | --- | --- | --- | --- | --- | --- | --- | --- | --- |
| Items | Study | N | b (p-value) | N | b (p-value) | N | b (p-value) | N | b (p-value) | N | b (p-value) |
| **MMSE** | **Total** | **1102** | **0.24 (<.001)** | **1100** | **0.02 (<.001)** | **1079** | **-0.34 (0.022)** | **1102** | **0.09 (<.001)** | 593 | 0.23 (0.545) |
|  | WF-ADRC | 283 | 0.23 (<.001) | 281 | 0.02 (0.027) | 278 | -0.24(0.224) | 283 | 0.09 (<.001) | . |  |
|  | DEMO | 116 | -0.07 (0.784) | 116 | 0.02 (0.644) | 116 | 0.03 (0.971) | 116 | 0.06 (0.552) | 114 | -1.79 (0.138) |
|  | HEALTHY | 39 | 0.28 (0.521) | 39 | -0.04 (0.485) | 39 | -0.15 (0.917) | 39 | 0.09 (0.628) | 38 | 2.51 (0.238) |
|  | IM-FIT | 148 | 0.45 (0.022) | 148 | 0.04 (0.081) | 145 | -0.71 (0.263) | 148 | 0.14 (0.103) | 132 | -1.27 (0.226) |
|  | INFINITE | 195 | 0.04 (0.776) | 195 | 0.01 (0.725) | 193 | -0.19 (0.685) | 195 | -0.04 (0.489) | . |  |
|  | LEAN | 26 | 0.68 (0.276) | 26 | 0.07 (0.380) | 26 | -2.53 (0.203) | 26 | -0.01 (0.962) | 23 | -1.68 (0.577) |
|  | OPTIMA | 87 | 0.35 (0.096) | 87 | 0.06 (0.024) | 87 | -0.28 (0.676) | 87 | 0.11 (0.223) | 87 | -0.47 (0.635) |
|  | PART | 25 | -0.26 (0.269) | 25 | -0.03 (0.360) | 22 | -0.19 (0.810) | 25 | -0.03 (0.771) | 25 | 0.36 (0.751) |
|  | PROMO | 14 | -0.10 (0.872) | 14 | -0.03 (0.763) | 14 | 1.35 (0.511) | 14 | 0.00 (0.999) | 13 | -3.76 (0.243) |
|  | RAIN | 70 | 0.50 (0.010) | 70 | 0.03 (0.235) | 64 | -2.23 (<.001) | 70 | 0.32 (<.001) | 66 | 2.51 (0.012) |
|  | SILVER | 23 | 0.42 (0.369) | 23 | 0.03 (0.649) | 23 | -1.15 (0.444) | 23 | 0.17 (0.407) | 23 | -0.24 (0.914) |
|  | SWALLW | 76 | 0.42 (0.002) | 76 | 0.04 (0.043) | 72 | -0.09 (0.869) | 76 | 0.10 (0.071) | 72 | 0.75 (0.249) |
|  |  |  |  |  |  |  |  |  |  |  |  |
| **MOCA** | **Total** | **504** | **0.31 (<.001)** | **502** | **0.04 (<.001)** | **494** | **-0.37 (0.032)** | **504** | **0.08 (0.008)** | 149 | -0.36 (0.631) |
|  | WF-ADRC | 287 | 0.32 (<.001) | 285 | 0.04 (<.001) | 282 | -0.26 (0.168) | 287 | 0.12 (<.001) | . |  |
|  | APPLE | 37 | 0.16 (0.729) | 37 | 0.03 (0.660) | 37 | -0.50 (0.621) | 37 | -0.01 (0.972) | . |  |
|  | EVIDNCE | 14 | 1.58 (<.001) | 14 | 0.14 (0.017) | 13 | -3.70 (<.001) | 14 | -0.09 (0.634) | 14 | -1.10 (0.571) |
|  | MEDFST | 138 | 0.24 (0.196) | 138 | 0.05 (0.048) | 134 | -0.43 (0.294) | 138 | -0.02 (0.747) | 135 | -0.24 (0.761) |
|  | OPTFST | 28 | -0.36 (0.438) | 28 | 0.03 (0.649) | 28 | -0.22 (0.825) | 28 | -0.34 (0.064) | . |  |
|  |  |  |  |  |  |  |  |  |  |  |  |
| **DSST** | **Total** | **584** | **0.75 (<.001)** | **577** | **0.08 (<.001)** | **561** | **-1.12 (<.001)** | **585** | **0.14 (<.001)** | 270 |  |
|  | WF-ADRC | 280 | 0.61 (<.001) | 278 | 0.08 (<.001) | 276 | -0.98 (<.001) | 280 | 0.09 (0.076) | . |  |
|  | MEDFST | 138 | 0.54 (0.007) | 138 | 0.05 (0.031) | 134 | -0.84 (0.032) | 138 | 0.11 (0.215) | 135 |  |
|  | OPTFST | 28 | 0.52 (0.175) | 28 | 0.08 (0.067) | 28 | -1.02 (0.169) | 28 | 0.04 (0.817) | . |  |
|  | PA-AML | 62 | 2.01 (<.001) | 57 | 0.16 (<.001) | 51 | -3.04 (<.001) | 63 | 0.48 (<.001) | 63 |  |
|  | SWALLW | 76 | 0.76 (0.001) | 76 | 0.07 (0.013) | 72 | -1.03 (0.026) | 76 | 0.24 (0.018) | 72 |  |

Note. Study name abbreviations are found in Appendix 1, SD= standard deviation, SPPB= Short Physical Performance Battery, m/s= meters per second, s= seconds, MMSE= Mini Mental Status Exam, DSST= Digit Symbol Substitution Test, MoCA= Montreal Cognitive Assessment, SPPB= Short Physical Performance Battery, m/s= meters per second, s= seconds. A balance score of 0 is a valid number, not a missing value. All balance score are from 0-4.

Supplementary Table 5. Regression Coefficient (SD unit) Adjusted for Age, Sex, Race, and Body Mass Index, in a sensitivity analysis of 4 studies with more than one cognitive function test

|  |  | **SPPB Score** |  | **4m Walk (m/s)** |  | **Chair Rise Time (s)** |  | **Balance Score** |  | **Grip Strength (kg)** |  |
| --- | --- | --- | --- | --- | --- | --- | --- | --- | --- | --- | --- |
| Items | Study | N | b (p-value) | N | b (p-value) | N | b (p-value) | N | b (p-value) | N | b (p-value) |
| **MMSE** | **Total** | **359** | **0.25 (<.001)** | **357** | **0.02 (0.028)** | 350 | -0.22 (0.118) | **359** | **0.09 (0.001)** | **72** | 0.49(0.456) |
|  | WF-ADRC | 283 | 0.22 (0.002) | 281 | 0.02 (0.097) | 278 | -0.24 (0.112) | 283 | 0.09 (0.004) | . |  |
|  | SWALLW | 76 | 0.41 (0.010) | 76 | 0.03 (0.084) | 72 | -0.08 (0.835) | 76 | 0.10 (0.120) | 72 | 0.49(0.456) |
|  |  |  |  |  |  |  |  |  |  |  |  |
| **MOCA** | TOTAL | **453** | **0.29 (<.001)** | **451** | **0.04 (<.001)** | 444 | -0.28 (0.112) | 453 | **0.08 (0.007)** | 135 | -0.28(0.729) |
|  | WF-ADRC | 287 | 0.31 (<.001) | 285 | 0.04 (<.001) | 282 | -0.25 (0.194) | 287 | 0.12 (<.001) | . |  |
|  | MEDIFAST | 138 | 0.24 (0.206) | 138 | 0.05 (0.062) | 134 | -0.43 (0.300) | 138 | -0.03 (0.713) | 135 | -0.28(0.729) |
|  | OPTIFAST | 28 | -0.37 (0.431) | 28 | 0.02 (0.693) | 28 | -0.24 (0.809) | 28 | -0.35 (0.058) | . |  |
|  |  |  |  |  |  |  |  |  |  |  |  |
| **DSST** | Total | **522** | **0.57 (<.001)** | **520** | **0.07 (<.001)** | 510 | **-0.97 (<.001)** | **522** | **0.10 (0.005)** | 207 | 1.02(0.094) |
|  | WF-ADRC | 280 | 0.56 (<.001) | 278 | 0.08 (<.001) | 276 | -1.00 (<.001) | 280 | 0.07 (0.081) | . |  |
|  | MEDFST | 138 | 0.52 (0.003) | 138 | 0.05 (0.034) | 134 | -0.83 (0.031) | 138 | 0.11 (0.155) | 135 | 0.57(0.457) |
|  | OPTFST | 28 | 0.51 (0.134) | 28 | 0.08 (0.069) | 28 | -1.01 (0.168) | 28 | 0.04 (0.770) | . |  |
|  | SWALLW | 76 | 0.73 (<.001) | 76 | 0.07 (0.013) | 72 | -1.05 (0.022) | 76 | 0.23 (0.008) | 72 | 1.69(0.069) |

Note. Study name abbreviations are found in Appendix 1, SD= standard deviation, SPPB= Short Physical Performance Battery, m/s= meters per second, s= seconds, MMSE= Mini Mental Status Exam, DSST= Digit Symbol Substitution Test, MoCA= Montreal Cognitive Assessment, SPPB= Short Physical Performance Battery, m/s= meters per second, s= seconds. A balance score of 0 is a valid number, not a missing value. All balance score are from 0-4.

| Appendix 1. Summary Table of 17 Clinical Studies from Wake Forest University Included in the Pooled Analysis | | | | | |
| --- | --- | --- | --- | --- | --- |
| Study Name | N | Age Range | Study Criteria | PI | IRB number |
| **WF-ADRC**  (Alzheimer’s Disease Research Center at Wake Forest) | 289 | 55+ | Cognitively normal, Mild Cognitive Impairment, or Alzheimer’s Disease | Suzanne Craft, PhD | IRB00025540 |
| **APPLE**  (Arthritis Pilot for Preserving Muscle While Losing Weight) | 37 | 65-80 | Overweight/obese; sedentary, diagnosis of osteoarthritis, cognitively normal | Barbara Nicklas, PhD | IRB00028928 |
| **DEMO**  (Diet, Exercise, and Metabolism in Older Women) | 116 | 50-70 | Women with abdominal obesity, postmenopausal, cognitively normal | Barbara Nicklas, PhD | BG01-508 |
| **EVIDNCE**  (Vitamin D Supplementation and Physical Function in Older Adults- Pilot Study) | 14 | 65-89 | At risk for disability, (SPPB <10), Vitamin D insufficient (serum 25(OH)D ≥ 10 to <25 ng/mL), cognitively normal | Denise Houston, PhD & Stephen Kritchevsky, PhD | IRB00011371 |
| **HEALTHY**  (Database of determinants of physical function in healthy older persons) | 39 | 60+ | Healthy, free of chronic disease | Dalane Kitzman, MD | IRB00002439 |
| **IM-FIT**  (Improving Muscle for Functional Independence Trial) | 148 | 65-80 | Obese, at risk for disability, cognitively normal | Barbara Nicklas, PhD | IRB00009098 |
| **INFINITE**  (Investigating Fitness Interventions in the Elderly) | 195 | 65-80 | Obese, sedentary, cognitively normal | Barbara Nicklas, PhD | IRB00008292 |
| **LEAN**  (Lean Muscle Function) | 26 | 65-75 | Normal weight, no resistance training for past 6 months, cognitively normal | Barbara Nicklas, PhD | IRB00019632 |
| **MEDFST**  (Effect of High Protein Weight Loss on physical function for Seniors) | 138 | 65-79 | Overweight/obese, sedentary, self-reported mobility disability, cognitively normal | Kristen Beavers, PhD, MPH | IRB00033428 |
| **OPTFST**  (Impact of Weight Loss on Physical Function) | 28 | 65+ | Obese (BMI greater than or equal to 35 kg/m2), cognitively normal | Jamy Ard, MD | IRB00025897 |
| **OPTIMA**  (Optimizing Body Composition for Function in Older Adults) | 87 | 65-80 | Overweight/obese, at risk for disability, cognitively normal | Stephen Kritchevsky, PhD | BG06-051 |
| **PA-AML**  (Symptom- Adapted Physical Activity Intervention in Minimizing Physical Function Decline in Older Patients With Acute Myeloid Leukemia Undergoing Chemotherapy) | 63 | 60+ | Diagnosis of Acute Myeloid Leukemia, no cognitive impairment | Heidi Klepin, MD | IRB00019467 |
| **PART**  (Physical Activity Program for Older Renal Transplant Candidates) | 25 | 60+ | Listed on the renal transplant waiting list, cognitively normal | Erica Hartman, MD; Stephen Kritchevsky, PhD | IRB00001451 |
| **PROMO**  (Dietary Protein & Body Composition in Older Women) | 14 | 50-75 | Overweight/obese, at risk for disability, cognitively normal | Stephen Kritchevsky, PhD | BG04-149 |
| **RAINS**  (Reducing Age Related Inflammation with Nutritional Supplementation) | 70 | 65-85 | Overweight/obese, at risk for disability, cognitively normal | Stephen Kritchevsky, PhD | IRB00003048 |
| **SILVER**  (Use of a Soy-based Meal Replacement Weight Loss Intervention to Impact Ectopic Fat) | 23 | 60-79 | Overweight/obese, cognitively normal | Mara Vitolins, DrPH & Kristen Beavers, PhD | IRB00015659 |
| **SWALLW**  (CT Imaging of Lingual Muscle Fat Composition in Community-Dwelling Older Adult Aspirators and Non Aspirators) | 76 | 65-90 | Healthy older adults | Susan Butler, PhD | IRB00005219 |
